# Blood vessel organoids generated by base editing and harboring single nucleotide variation in Notch3 effectively recapitulate CADASIL-related pathogenesis

**DOI:** 10.1007/s12035-024-04141-4

**Published:** 2024-04-09

**Authors:** Yujin Ahn, Ju-Hyun An, Hae-Jun Yang, Wi-Jae Lee, Sang-Hee Lee, Young-Ho Park, Jong-Hee Lee, Hong J. Lee, Seung Hwan Lee, Sun-Uk Kim

**Affiliations:** 1 Futuristic Animal Resource and Research Center, Korea Research Institute of Bioscience and Biotechnology, Ochang, Chungcheongbuk-do 28116 Korea; 2 Department of Functional Genomics, KRIBB School of Bioscience, Korea University of Science and Technology, Daejeon, 34113 Korea; 3 Department of Life Science, Chung-Ang University, Seoul, 06974 Republic of Korea; 4 Center for Research Equipment (104-Dong), Korea Basic Science Institute, Ochang, Cheongju, Chungbuk 28119 Republic of Korea; 5 National Primate Research Center (NPRC), Korea Research Institute of Bioscience and Biotechnology (KRIBB), Ochang, 28116 Korea; 6 College of Medicine and Medical Research Institute, Chungbuk National University, Cheongju, 28644 Korea; 7 Research Institute, huMetaCELL Inc., Gyeonggi-do, Korea; 8 Department of Biomedical Engineering, University of Alabama at Birmingham, Birmingham, United States; 9 Department of Neurosurgery, University of Alabama at Birmingham, Birmingham, United States

**Keywords:** Human blood vessel organoids, CADASIL, NOTCH3 gene, CRISPR/Cas9 base editing, Mural cell degeneration

## Abstract

Human blood vessel organoids (hBVOs) offer a promising platform for investigating vascular diseases and identifying therapeutic targets. In this study, we focused on *in vitro* modeling and therapeutic target finding of cerebral autosomal dominant arteriopathy with subcortical infarcts and leukoencephalopathy (CADASIL), the most common form of hereditary stroke disorder caused by mutations in the NOTCH3 gene. Despite the identification of these mutations, the underlying pathological mechanism is elusive, and effective therapeutic approaches are lacking. CADASIL primarily affects the blood vessels in the brain, leading to ischemic strokes, migraines, and dementia. By employing CRISPR/Cas9 base-editing technology, we generated human induced pluripotent stem cells (hiPSCs) carrying Notch3 mutations. These mutant hiPSCs were differentiated into hBVOs. The NOTCH3 mutated hBVOs exhibited CADASIL-like pathology, characterized by a reduced vessel diameter and degeneration of mural cells. Furthermore, we observed an accumulation of Notch3 extracellular domain (Notch3ECD), increased apoptosis, and cytoskeletal alterations in the NOTCH3 mutant hBVOs. Notably, treatment with ROCK inhibitors partially restored the disconnection between endothelial cells and mural cells in the mutant hBVOs. These findings shed light on the pathogenesis of CADASIL and highlight the potential of hBVOs for studying and developing therapeutic interventions for this debilitating human vascular disorder.

**Supplementary Information:**

The online version contains supplementary material available at 10.1007/s12035-024-04141-4.

## Introduction

Cerebral autosomal dominant arteriopathy with subcortical infarcts and leukoencephalopathy (CADASIL) is the most prevalent hereditary stroke disorder. The condition arises from mutations in the NOTCH3 gene on chromosome 19p13.1 [1, 2]. The main symptoms are migraines, psychiatric disorders, recurrent strokes, and dementia [3]. Notch3 mutation is associated with molecular pathologies such as arterial smooth muscle degeneration and the occurrence of granular osmiophilic material (GOM) [4]. However, the mechanism of pathogenesis is unclear, and there is no effective therapeutics for CADASIL.

The NOTCH3 gene encodes the Notch3 receptor, a single-pass transmembrane receptor predominantly expressed in mural cells, including vascular smooth muscle cells (VSMCs) and pericytes [5, 6]. The Notch3 receptor consists of 29–36 epidermal growth factor repeats (EGFRs) in its extracellular domain [7]. Variants that alter the number of cysteine residues in the EGFRs contribute to the CADASIL pathogenesis [8]. An odd number of cysteine residues leads to the formation of incomplete disulfide bridges, increasing the multimerization potential between Notch3 ectodomains (Notch3ECD) [9]. Moreover, mutations in the Notch3 receptor result in mural cell degeneration, accumulation of Notch3ECD, and the deposition of GOM [9–11]. However, the relationship between NOTCH3 mutations and the pathophysiological symptoms is poorly understood.

In this study, we generated a novel CADASIL model using human blood vessel organoids (hBVOs), which represent human blood vessel-like structures and enable the investigation of vascular cell interactions at both the cellular and tissue levels [12, 13]. Using clustered regularly interspaced short palindromic repeats (CRISPR)/Cas9 base-editing technology, we generated human induced pluripotent stem cells (hiPSCs) carrying Notch3 mutations, free from other background influences, such as sporadic or inherited genetic aberrations. These genetically engineered hiPSCs were differentiated into hBVOs. The Notch3-mutated hBVOs exhibited decreased vessel diameter and loss of mural cells. Additionally, the levels of notch3ECD and apoptosis were increased in Notch3 mutant BVOs. Furthermore, cytoskeleton alterations were detected in Notch3 mutant BVOs. To identify factors capable of restoring connections between vascular cells, we conducted an inhibitor treatment study. The results revealed that the Rho kinase (ROCK) inhibitor partially restored intercellular connectivity and protected mural cells from degeneration. This platform holds promise for elucidating pathophysiological mechanisms and identifying novel target drugs for human vascular disease, especially for CADASIL.

## Results

### Generation and Characterization of Notch3 Mutant hiPSCs

**Fig. 1:**
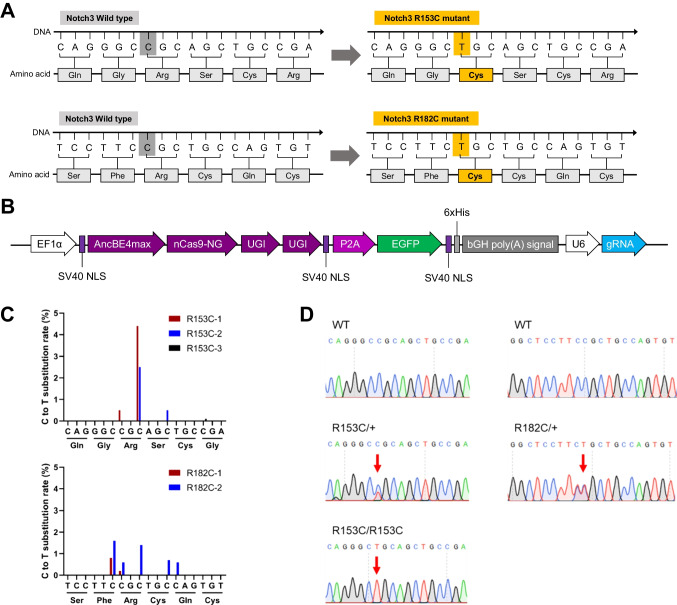
CRISPR base editing for Notch3 mutation. (**A**) Schematic representation of missense mutations (R153C or R182C) in the Notch3 gene. (**B**) Overview of vector structure of all-in-one CRISPR base editor for Notch3 mutation. (**C**) C to T substitution rate at the targeted loci in HEK293 cells. (**D**) Sanger sequencing of base editing target loci in wild type (WT) and designated Notch3 mutations in established hiPSC lines

The missense Notch3 mutant responsible for CADASIL is characterized by a single nucleotide change that results in the alteration of cysteine residues [14]. We aimed to introduce the R153C or R182C Notch3 mutation by inducing a cytosine-to-thymine (C-T) conversion using CRISPR/Cas9 base editing technology, resulting in an amino acid substitution from arginine to cysteine (Fig. 1A). Specific sgRNA candidates were selected to recognize the target loci of NOTCH3 gene to induce the R153C or R182C mutations, which direct the Cas9 nickases (nCas9) to induce single-strand breaks (Table 1). The conversion of targeted single nucleotide is enabled by CRISPR base editing [15]. For better accessibility to target loci, we constructed base editor using nCas9-NG, which can recognize NG protospacer adjacent motif (PAM) sequences [16]. The base editor AncBE4max was adopted to induce a C-T conversion [17]. The customized base editor, EF1α-AncBE4max-nCas9-2UGI-EGFP-U6-sgRNA plasmid vector was transfected into HEK293 cells to test its target base conversion efficiency (Fig. 1B). Following transfection, the plasmids containing sgRNAs (R153C-1, R182C-1, and R182C-2) exhibited target C-T base substitution efficiencies of 0.5%, 0.2%, and 0.5%, respectively (Fig. 1C).

Wild-type (WT) human induced pluripotent stem cells (hiPSCs) were transfected with the base editor plasmids containing the most efficient sgRNAs (R153C-1 and R182C-1), and EGFP-expressing hiPSCs were isolated by fluorescence activated cell sorting (FACS). Among the EGFP-expressing hiPSC single cell colonies, we identified the several iPSC clones that harbor the intended R153C homozygous, R153C heterozygous, and R182C heterozygous mutations (Fig.1D).

**Fig. 2:**
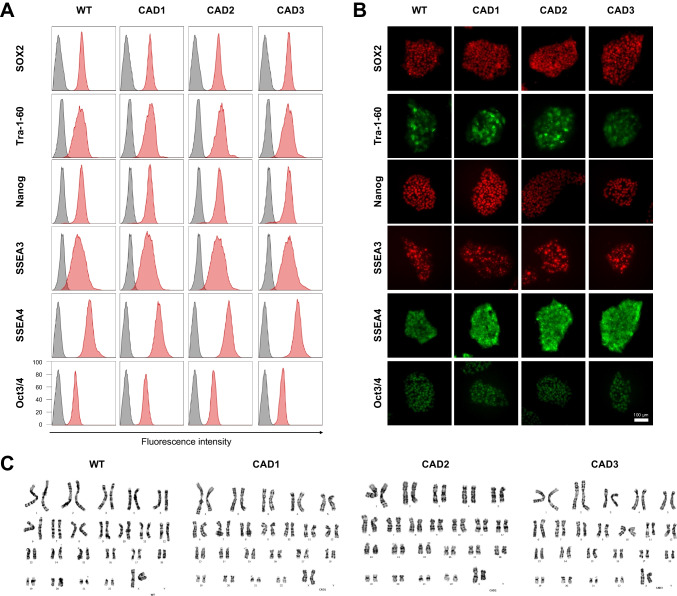
Characterization of Notch3 mutant hiPSCs (**A**) Flow cytometry of the pluripotency markers SOX2, Tra-1-60, Nanog, SSEA3, SSEA4, and Oct3/4. Isotype control (gray), hiPSCs (red). CAD1 (R153C heterozygous), CAD2 (R153C homozygous), CAD3 (R182C heterozygous). (**B**) Immunostaining of the pluripotency markers SOX2, Tra-1-60, Nanog, SSEA3, SSEA4, and Oct3/4. Scale bar, 100 μm. (**C**) Karyotype analysis demonstrating a normal diploid female (46, XX) karyotype of wild type (WT) and Notch3 mutant hiPSC lines.

We assessed the pluripotency and genomic stability of the gene-edited Notch3 mutant hiPSCs. The cell lines expressed pluripotency markers, including SOX2, Tra-1-60, Nanog, SSEA3, SSEA4, and Oct3/4, as confirmed by flow cytometry and immunostaining (Fig. 2A and 2B). Additionally, a normal karyotype of (46, XX) was observed in the analyzed cells (Fig. 2C). To identify any unintended mutations resulting from gene editing, an off-target analysis was conducted. The top 10 off-target sites were predicted using the benchling web tool (Table 2 and Table 3). Sanger sequencing was performed for the top 10 predicted off-target sites around each sgRNA (R153C-1 and R182C-1) (Supplementary Fig. 1A and 1B). Notably, no mutation was detected in any of the off-target sequences, suggesting that off-target cleavage is unlikely to have contributed to CADASIL-like pathology.

### Differentiation of hBVOs and Vascular Structural Changes in Notch3 Mutant hBVOs

**Fig. 3:**
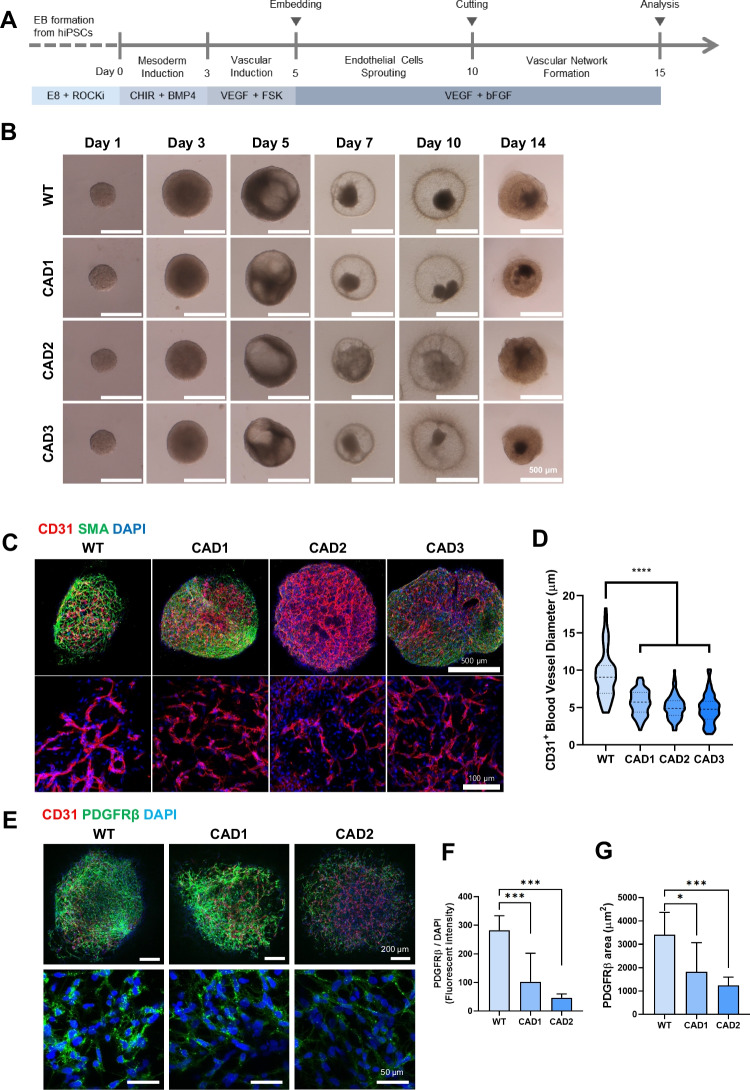
Generation of and structural differences in Notch3 mutant hBVOs. (**A**) Schematic diagram of the generation of hBVOs. (**B**) Representative images of WT and Notch3 mutant hBVOs. CAD1 (R153C heterozygous), CAD2 (R153C homozygous), CAD3 (R182C heterozygous). Scale bar, 500 µm. (**C**) CD31^+^ endothelial networks in WT and Notch3 mutant hBVOs. (**D**) CD31^+^ blood vessel diameters in WT and Notch3 mutant hBVOs. Scale bar, 500 µm and﻿ 100 µm.﻿ (**E**) Reduced CD140b (PDGFRβ)^+^ pericytes in Notch3 mutant hBVOs. Scale bar, 200 µm and﻿ 50 µm.﻿ (**F**) Significantly decreased fluorescence intensity and area of CD140b (PDGFRβ) signals in Notch3 mutant hBVOs. Data are means ± SEMs. *p < 0.05, ***p < 0.001, ****p < 0.0001

To investigate the effects of NOTCH3 mutations on vascular structure, Notch3 mutant hiPSCs were differentiated into hBVOs using a mesoderm induction and vascular differentiation protocol [13] for 15 days (Fig. 3A). Visual observation under the microscope confirmed the presence of well-established blood vessel-like structures in both WT and Notch3 mutant hBVOs (Fig. 3B).

To examine the differences in the vasculature between WT and Notch3 mutant hBVOs, immunostaining for CD31^+^ endothelial cells and SMA^+^ smooth muscle cells was performed in each group. As a result, we found that the diameter of CD31^+^ endothelial vessels was significantly decreased in Notch3 mutant hBVOs compared to WT (Fig. 3C and 3D). Furthermore, PDGFRβ expression was significantly decreased, with heterozygous CADASIL hBVOs showing a 2.7-fold reduction and homozygous CADASIL hBVOs showing a 6-fold reduction compared to WT hBVOs (Fig. 3E and 3F). In addition, PDGFRβ positive area significantly was decreased in CADASIL hBVOs (Fig. 3G). These results suggest that Notch3 mutant hBVOs exhibit a phenotype characterized by reduced blood vessel diameter and loss of PDGFRβ expression, which recapitulates functional impairments observed in blood vessel of CADASIL patients [17].

### Increased Apoptosis and Altered Cytoskeleton in Notch3 Mutant hBVOs

**Fig. 4:**
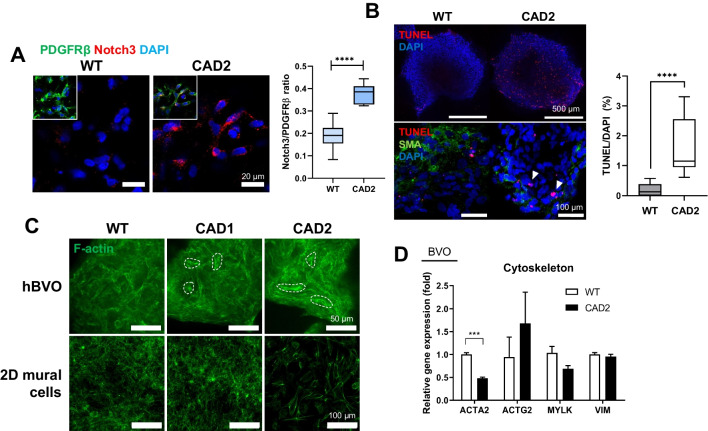
Main pathologic characteristics of CADASIL in Notch3 mutant hBVOs. (**A**) Increased expression of Notch3ECD in Notch3 mutant hBVOs. (**B**) Increased apoptotic signals in Notch3 mutant hBVOs. (**C**) Alteration of cytoskeleton morphology in Notch3 mutant hBVOs (hBVO) and 2D-differentiated mural cells (2D mural cells). The actin filament nodes were highlighted by dashed lines. (**D**) qRT-PCR result of cytoskeleton-associated genes in WT and Notch3 mutant hBVOs. CAD1 (R153C heterozygous), CAD2 (R153C homozygous). Data are means ± SEMs. ***p < 0.001, ****p < 0.0001.

We next investigated the CADASIL specific key characteristics, including the accumulation of Notch3ECD and the presence of granular osmiophilic material (GOM) in Notch3 mutant hBVOs. To examine the alteration in Notch3ECD expression, WT and Notch3 mutant hBVOs were immunolabeled using anti-Notch3ECD and anti-PDGFRβ antibodies. The relative abundance of Notch3ECD per total mural cells was significantly higher in Notch3 R153C homozygous mutant hBVOs (Fig. 4A). This indicates the accumulation of Notch3ECD in Notch3 mutant hBVOs despite a decrease in Notch3 receptor-expressing mural cells.

Another key vascular pathological hallmark of CADASIL is the accumulation of GOM on the periphery of the mural cells. We adopted transmission electron microscopy (TEM) to detect GOM ultrastructure in Notch3 mutant hBVOs. We successfully identified endothelial cells and surrounding extracellular matrix, however, there was no GOM ultrastructure deposits in all specimen we analyzed (Supplementary Fig. 2).

Next, we investigated the reasons for the degeneration of mural cells in Notch3 mutant hBVOs. We examined the alterations in the differentiation and proliferation potential of mural cells derived from Notch3 mutant hiPSCs (Supplementary Fig. 3A). Interestingly, there was no significant difference in the cellular proportion of PDGFRβ-positive mural cells between WT and Notch3 mutant hiPSCs after differentiation (Supplementary Fig. 3B). Additionally, there was no significant disparity in the cell cycle phase distribution between WT and Notch3 R153C homozygous mural cells, as determined by propidium iodide (PI) staining (Supplementary Fig. 3C and 3D). On the other hand, TUNEL assay revealed that apoptotic cells were significantly increased in Notch3 R153C homozygous hBVOs (Fig. 4B). Therefore, the degeneration of mural cells in Notch3 mutant hBVOs is not attributable to their differentiation or proliferation potential, but to increased apoptosis.

Moreover, the morphology of the cytoskeleton was distinguishably altered both in Notch3 R153C homozygous hBVOs and mural cells derived from the same cell line with the 2-dimensional (2D)-differentiation protocol (Fig. 4C). In Notch3 R153C homozygous hBVOs, filamentous actin formed bundled node structures, which is prevalently observed in CADASIL patient blood vessels [3]. Concordantly, 2D-differentiated Notch3 R153C homozygous mural cells also exhibited disorganized cytoskeletal nodes and elongated filamentous actin structure, which is concurrently reported in previous articles that adopted 2D culture system [18]. Furthermore, expression of the ACTA2 gene, which is one of the cytoskeleton-associated genes, was downregulated in Notch3 R153C homozygous hBVOs (Fig. 4D).

In summary, our data demonstrated that mural cell degeneration in Notch3 mutant BVOs is not influenced by their differentiation and proliferation potential. Instead, Notch3 mutations appear to aggravate mural cells dysfunction, as evidenced by increased TUNEL signals within organoids. Furthermore, we identified the altered cytoskeletal structures in both Notch3 mutant hBVOs and 2D-cultured mural cells, suggesting that Notch3 mutations contribute to mural cell dysfunction. Overall, Notch3 mutations lead to vascular dysfunction through increased apoptosis and disorganized cytoskeleton in mural cells.

### Restoration of Vascular Cell Interactions by a ROCK Inhibitor

**Fig. 5:**
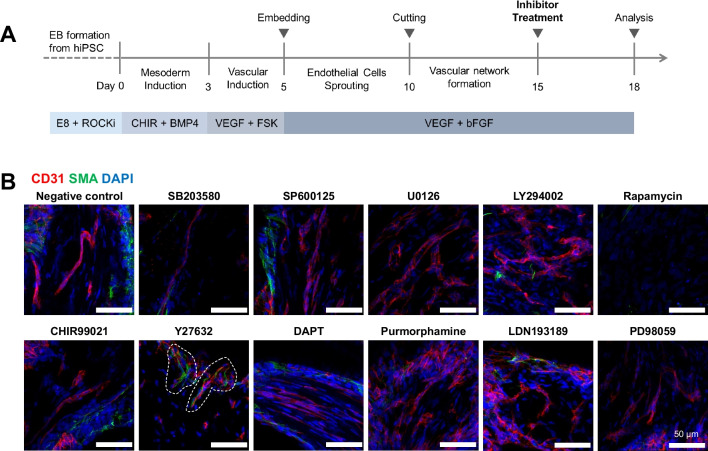
Restoration of the interaction between vascular cells by a ROCK inhibitor**.** (**A**) Schematic diagram for inhibitor study using Notch3 mutant hBVOs. (**B**) Immunofluorescence images showing SMA^+^ mural cells (green) and CD31^+^ endothelial cells (red) in Notch3 R153C homozygous hBVOs treated with small chemicals targeting different key signaling pathways as indicated in the figure. Dashed lines indicate the region that intercellular connections are recovered

In search of potential therapeutic targets for CADASIL, we explored the effects of various inhibitors and activators on vasculopathies in hBVOs. From day 15 to 18 of hBVO differentiation, Notch3 R153C homozygous hBVOs were treated with inhibitors targeting key signaling pathways, including p38, c-Jun N-terminal kinase (JNK), extracellular signal-regulated kinase (ERK), Protein kinase B (AKT), Mitogen-activated protein kinase kinase (MEK1/2), glycogen synthase kinase 3 beta (GSK3β), mammalian target of rapamycin (mTOR), Rho-associated coiled-coil containing kinase (ROCK), γ-secretase, sonic hedgehog (SHH), and transforming growth factor-beta (TGF-β), at known physiologically active concentrations [12, 19–29] (Fig. 5A). As previously described, Notch3 mutant hBVOs exhibited impaired intercellular connection between endothelial cells and mural cells. Among all candidates, only the ROCK inhibitor restored the interaction between mural cells and endothelial cells, as evidenced by the recovered co-localization of mural cells with endothelial cells after treatment (Fig. 5B). Although the restoration did not reach the level in WT samples, mural cells reestablished connections with endothelial cells. These findings highlight the potential of Notch3 mutant hBVOs as a platform for investigating targeted drug therapies for CADASIL.

## Discussion

In this study, we established a robust CADASIL model using hBVOs derived from Notch3 mutant hiPSCs. We utilized CRISPR/Cas9 base-editing strategy to introduce the most prevalent CADASIL-associated NOTCH3 genetic mutations, R153C or R182C [14, 30], into hiPSCs. This approach ensured a controlled genetic background that allows us to validate the consequential effects of Notch3 mutation on CADASIL pathogenesis by excluding other pathogenic mutations. Introduction of desired Notch3 single nucleotide variants was efficiently achieved by using specific sgRNAs and the nCas9-NG [16] linked AncBE4max [17]. As a result of CRISPR base editing, we obtained multiple Notch3 gene mutant hiPSC lines, which harbor desired genetic mutations on the target loci (Fig. 1 and 2). These hiPSC clones enabled us to investigate the impact of different types of CADASIL-associated Notch3 variants on human vasculogenesis. The genetically engineered hiPSCs were subsequently differentiated into hBVOs to investigate the vascular pathologies that are associated with CADASIL.

Characterization of the Notch3 mutant hBVOs revealed several key features reminiscent of the vascular pathology of CADASIL. The Notch3 mutant hBVOs exhibited a decreased diameter of endothelial vessels and a significant decrease in the population of PDGFRβ-positive mural cells compared to WT hBVOs. These findings provide evidence that the Notch3 mutant hBVOs accurately recapitulate the vascular abnormalities shown in CADASIL patients [3]. Furthermore, our Notch3 mutant hBVO model exhibited the distinguishable cytoskeletal node formation (Fig. 4) which evidences the consequential effect of Notch3 mutation on CADASIL-related cytoskeleton disorganization in VSMCs [31]. These results were concordant with previous reports employing CADASIL patient cells or patient cell-derived iPSCs [32, 33], suggesting that our model faithfully recapitulates CADASIL-related pathologies. These findings highlight the significance of hBVOs as a surrogate model for CADASIL, elucidating the multifaceted nature of its pathophysiological manifestations.

To further explore the pathological characteristics of CADASIL hBVOs, we focused on the accumulation of Notch3ECD and GOM. Immunolabeling revealed a significantly increased ratio of Notch3ECD expression per total mural cells in the Notch3 R153C homozygous mutant hBVOs. While the abundance of PDGFRβ-expressing mural cells, which are responsible for Notch3 expression, was decreased in Notch3 mutant hBVOs, Notch3ECD accumulation was significantly increased. These findings provide evidence that Notch3 mutation impairs the balance in Notch3 shedding and processing. To detect GOM ultrastructure deposition in hBVOs, we performed TEM. Although we obtained clear endothelial cell images using TEM, we could not identify GOM deposition from any specimen we analyzed. The previous report that adopted CADASIL patient iPSCs as a disease model demonstrated that 2D-differentiated CADASIL VSMCs exhibit the latent-transforming growth factor beta-binding protein-1 (LTBP-1) or high temperature requirement A1 (HtrA1) deposition, which are known components of GOM [18]. The reason why our Notch3 mutant hBVO model does not exhibit GOM deposition can be attributed to two factors. Since the causal factor of GOM is not fully elucidated, our data imply that Notch3 mutation alone may not be sufficient to induce this pathology. Secondly, the capillary-like nature of hBVOs’ vasculature may not be ideal for detecting the GOM deposits. In the CADASIL patients and mouse models, GOM deposits are most prominent in small to medium-sized arteries within the brain [34], whereas the vasculatures of hBVOs primarily consists of a capillary vascular structure [13]. Therefore, inducing directed differentiation of organoids towards an arteriolar lineage could be a promising approach to model GOM pathology.

To identify therapeutic targets for CADASIL, a range of inhibitors and activators targeting various signaling pathways were tested in the Notch3 mutant hBVOs. Among all candidates, Y27632, a ROCK signaling inhibitor, only restored the interconnection between mural and endothelial cells. In the context of human vascular structure homeostasis, Rho kinase signaling plays a key role in vascular dysfunction and senescence [35], which is governed by the Notch signaling cascade [36]. In VSMCs, the NOTCH pathway activation leads to increased endoplasmic reticulum (ER) stress and Rho kinase activity [31, 37], thereby contributing to vascular dysfunctions, such as cytoskeleton disorganization, increased susceptibility, and impairment of contractibility. Additionally, Notch signaling is responsible for the intercellular communication between VSMCs and endothelial cells [38]. In our results, while the restoration of vasculature did not reach the level observed in WT samples, it is noteworthy that ROCK inhibitor holds therapeutic potential for ameliorating the vascular pathology in CADASIL. As the pathologic phenotypes we observed on BVOs, including mural cell degeneration and cytoskeleton disorganization, are closely associated with abnormal activation of ROCK signaling [31], ROCK inhibitor potentially contributes to ameliorate those pathologies by controlling NOTCH3 mutation-mediated Rho kinase hyperactivation. However, despite these observations, the exact underlying mechanism of the therapeutic effect of the ROCK inhibitor remains unclear. Finally, given that our CADASIL hBVO model exhibited known NOTCH3 signaling-driven vasculopathies, such as actin filament node formation, increased apoptosis, mural cell degeneration, and impaired endothelial-mural cell connections, it will serve as a valuable research platform for investigating CADASIL pathogenesis and conducting drug screening.

In conclusion, we generated a robust CADASIL model using Notch3 mutant hiPSCs and hBVOs. Characterization of the mutant hBVOs revealed NOTCH3 mutant-associated vascular abnormalities, including cytoskeletal alterations, altered Notch3ECD expression, reminiscent of the CADASIL pathology. The identification of ROCK signaling inhibition as a potential therapeutic strategy highlights the utility of the hBVO model for investigating pathophysiological mechanisms and identifying novel target drugs for CADASIL and other human vascular diseases. Continued research utilizing the Notch3 mutant hBVO platform will provide further insight into CADASIL pathogenesis and will promote the development of effective treatments for this debilitating condition.

## Methods and Materials

### Design of sgRNAs

To target the regions R153C and R182C of the human NOTCH3 gene, sgRNAs were designed. The Benchling web tool (https://www.benchling.com/) was used to select the optimal target sites for the CRISPR-Cas9 base editing system. Five sgRNAs were used in subsequent experiments (Table 1). The sgRNAs were ligated to a backbone plasmid containing the U6 promoter, which was derived from the pSpCas9(BB)-2A-GFP plasmid (Addgene plasmid #48138). The U6-sgRNA construct was amplified by PCR for further use.

#### Construction of a Base-Editing Vector

The base editor was created by assembling components from multiple plasmids. First, the AncBE4max gene was isolated from the pCMV-AncBE4max-P2A-GFP vector (Addgene plasmid #112100), and the nSpCas9-NG gene was obtained from the pSI-Target-AID-NG plasmid (Addgene plasmid #119861). The AncBE4max gene was fused to the 5'-end of the nSpCas9-NG gene using the In-Fusion® HD cloning kit (TaKaRa). To generate the final construct, the EF1α-AncBE4max-P2A-GFP construct was fused to the U6-sgRNA construct [16], resulting in the all-in-one CRISPR base editor plasmid.

#### Base-Editor Efficiency Test Using Targeted Next Generation Sequencing (NGS)

HEK293 cells were maintained in DMEM containing 5% fetal bovine serum (FBS) medium at 37°C with 5% CO_2_. Cells were seeded onto 12-well plates at a density of 5 × 10^5^ cells per well and allowed to attach and grow for 16–24 hours prior to transfection. For transfection, the following conditions were employed: Lipofectamine 3000 (Thermo Fisher Scientific) was used at 3 μL, and the base editor plasmid containing the sgRNA sequence was added at 1.2 μg per reactant. The transfection mixture was prepared by combining and diluting these components with Opti-MEM to a total of 106 μL, following the manufacturer's protocol. After 2–3 days, GFP-expressing cells were sorted using flow cytometry, and replated to fresh cell culture vessels. Genomic DNA of the sorted cells were isolated and subjected to targeted NGS to analyze genetic alterations in target loci. Genomic DNA of GFP positive cells was amplified by two-step PCR. For the first PCR (adapter PCR), specific staggered primers were used to amplify the integrated fragments; Forward primer: ACACTCTTTCCCTACACGACGCTCTTCCGATCTGATGGACGCTTCCTCTGCTC, Reverse primer: GTGACTGGAGTTCAGACGTGTGCTCTTCCGATCTTTACGGCATGGTGAGGGTGC. For the second PCR (index PCR), Illumina barcoded sequences were added to distinguish the samples. The amplicons were analyzed using Illumina MiniSeq sequencing system; Forward primer: AATGATACGGCGACCACCGAGATCTACACTATAGCCTACACTCTTTCCCTACACGAC, Reverse primer: CAAGCAGAAGACGGCATACGAGATCGAGTAATGTGACTGGAGTTCAGACGTGT.

#### Off-target Analysis

Base editing can generate nonspecific and unintended genetic modifications due to mismatch tolerance. To characterize potential off-target effects in the hiPSCs, we selected top-ranking off-target exonic sites in the human genome using the benching web tool (Table 1). We performed targeted sequencing of the top 10 potential off-target sites from control, R153C monoallelic, and R153C biallelic hiPSC lines (Supplementary Fig. 1A and 1B). None of the off-target sequences had mutations, indicating that off-target cleavage was unlikely to have contributed to CADASIL-like pathology.

#### Maintenance of Feeder-Independent hiPSCs

Cell culture plates were coated with a Matrigel matrix (Corning, 354234) dissolved in KO-DMEM (Gibco, 10829018) to a final concentration of 0.3 mg/mL. Mouse embryonic fibroblast (MEF)-free hiPSCs were cultured in E8 medium on the matrix-coated plates. Passaging of hiPSCs was performed using ReLeSR (Stemcell Technology, ST05872) every 4–5 days.

#### Generation of Spin Embryoid Bodies

MEF-independent hiPSCs were washed twice with DPBS to remove debris and remaining medium. The hiPSCs were dissociated by adding 1 mL of Accutase (Stemcell Technologies, 07922) to each well and incubating for 3–5 minutes at 37°C. The detached hiPSCs were filtered using a 70 μm strainer and resuspended in 9 mL of KO-DMEM and transferred to a 15 mL tube. The tube was centrifuged at 1,200 rpm for 2 minutes, and the medium was aspirated. The hiPSCs were resuspended in 1 mL of E8 medium containing 5 μM Y-27632 (MCE, HY-10583). For manual cell counting, 10 μL of cell suspension were mixed with 10 μL of 0.1% Trypan blue solution in an extra-flat 96-well plate. A hemocytometer (Merck, Z359629) was used to enumerate live cells under a microscope. The desired number of cells (2,000 hiPSCs) was transferred to each well of an ultra-low attachment 96-well plate, and the volume was adjusted accordingly. To each well, 50 μL of E8 medium with 5 μM Y-27632 were added after transferring hiPSCs. The plate was centrifuged at 1500 rpm for 5 minutes and incubated overnight at 37°C.

#### Generation of Human Blood Vessel Organoids

To differentiate the spin embryoid bodies (2,000 hiPSCs each) into hBVOs, the spin embryoid bodies incubated in vascular organoid differentiation medium. The medium consisted of a 1:1 mixture of DMEM-F12 (Gibco, 11320033) and neurobasal medium supplemented with B27 supplement (Gibco, 12587010), N2 supplement (Gibco, 17502048), 1 mM GlutaMax (Gibco, 35050061), 1% penicillin-streptomycin, and 55 nM 2-mercaptoethanol. The spin embryoid bodies were treated with 12 μM CHIR99021 (Tocris, 4423) and 30 ng/mL BMP4 (Peprotech, 120-05) from day+0 to 2. On day+3 to 4, the medium was replaced to a fresh medium containing 100 ng/mL VEGF-A (Peprotech, 100-20) and 30 ng/mL forskolin (Stemgent, 04-0025). On day+5, the organoids were embedded in a 1:2 mixture of phenol red-free Matrigel (Corning, 356231) and collagen I (Advanced BioMatrix, 5005) solution. The embedded organoids were then incubated with StemPro34 medium (Gibco, 10639011) containing 5% inactivated FBS, 100 ng/mL VEGF-A, and 100 ng/mL FGF-2 (R&D Systems, 233-FB/CF). The medium was replenished every other days throughout the differentiation period. On day+7 or 8, endothelial cells sprouted from the aggregates, and vascular networks were established. On day+10, individual BVOs were isolated from the gel and transferred to 96-well ultra-low attachment round bottom plates for further maintenance until sampling.

#### Whole-Mount Immunostaining of Organoids

The organoids were subjected to immunofluorescence staining. First, the organoids were rinsed twice with phosphate-buffered saline (PBS) and treated with cell recovery solution (Corning, 354253) at 4°C for 1 hour to eliminate non-specific signals caused by Matrigel. After the PBS rinses, the organoids were fixed in 4% paraformaldehyde at room temperature (RT) for 1 hour and washed with PBS for a minimum of 30 minutes. The organoids were cleared using the CytoVista™ 3D Culture Clearing Kit (Invitrogen, MAN0017942), following the manufacturer’s instructions. All procedures were carried out on a shaker. The organoids were subjected to a gradient series of methanol (50%, 80%, and 100%) at 4°C to achieve permeabilization. Subsequently, they were washed at RT using a series of methanol (80%, 50%, PBST, and PBS). The samples were next immersed in the antibody penetration buffer at RT for 1 hour and blocked with blocking buffer at 37°C. Primary antibodies were diluted in antibody dilution buffer, and the samples were incubated overnight at 37°C in the antibody solution. Next, the organoids were washed five times for 10 minutes each in a washing buffer and incubated overnight at 37°C with a diluted solution of secondary antibody and DAPI. Subsequently, the samples were washed 10 times for 10 minutes in the washing buffer. Samples were dehydrated using increasing concentrations of methanol (50%, 80%, and 100%) and incubated in the CytoVista tissue clearing reagent overnight at 4°C. Z-stack imaging was performed using a confocal microscope.

#### Quantitative Real-Time Reverse-Transcription PCR (qRT-PCR)

Samples were washed with PBS and immediately flash-frozen in liquid nitrogen. Total RNA was extracted using the RNeasy Mini Kit (Qiagen), and cDNA was synthesized from the RNA using random primers and reverse transcriptase (Toyobo). qRT-PCR was performed on a real-time PCR system (Thermo Fisher Scientific), and relative mRNA quantification was determined using the 2∆∆CT method.

#### Mural Cell Differentiation

Mural cell differentiation was carried out using a neuroectodermal intermediate differentiation protocol as described in a previous study [39]. hiPSCs were dissociated using ReleSR and plated onto Matrigel-coated six-well plates at a density of approximately 30,000 cells per well in E8 medium supplemented with 10 μM Y-27632. After 24 hours, the culture medium was replaced to fresh E8 medium. The following day, the cells were cultured in E6 medium containing 10 μM SB-431542 and 20 ng/mL FGF2. The medium was refreshed daily until day 6 of differentiation when the supplements were replaced with 2 ng/mL TGF-β and 5 ng/mL PDGF-BB. Daily medium changes were performed until day 18 of differentiation.

#### Flow Cytometric Analysis

To assess the proportions of cell types in hBVOs, flow cytometry was performed. hBVOs were dissociated using collagenase B for 30–40 minutes. The dissociated cells were pipetted and washed in PBS supplemented with 5% FBS. Subsequently, single cells were stained with fluorescence-conjugated antibodies for 30 minutes at 4°C. For intracellular antigen staining, cells were fixed using 4% paraformaldehyde and permeabilized with 0.1% saponin. Antibodies (CD31 and CD140b), diluted in PBS containing 5% FBS, were used to stain the cells. Flow cytometry data were acquired using the BD FACS Aria flow cytometer and analyzed using FlowJo software.

#### Preparation of Samples for TEM

To visualize GOM in Notch3 mutant hBVOs, we followed a previous protocol [40] for sample preparation and imaging using TEM. hBVO samples were fixed in a solution of 2% glutaraldehyde and 2% paraformaldehyde in 0.1 M phosphate buffer. Subsequently, the samples were postfixed in buffered osmium tetroxide. Dehydration of the samples was performed using a series of graded alcohols. Finally, the samples were embedded in an Epon-Araldite mixture. Semi-thin sections (2 μm) were obtained from the embedded samples using a microtome and stained with toluidine blue for examination. Thin sections were cut from the blocks using an ultramicrotome and stained with lead citrate for TEM imaging. The stained samples were carefully examined using TEM to visualize the presence of GOM. This process enabled capture of high-resolution images of GOM in Notch3 mutant hBVOs.

#### Propidium Iodide (PI) Staining

After differentiation, mural cells were fixed overnight at -20°C in 70% ethanol. Following two washes with PBS, the cells were incubated with 50 μg/mL PI (Thermo Fisher) in the dark at room temperature for 15 minutes in the presence of 100 μg/mL RNase A (Thermo Fisher). Subsequently, the cells were analyzed using flow cytometry to determine the cell cycle distribution.

#### Inhibitor Study

The following inhibitors or activators were used: SB203580 for p38 inhibition (10 μM; Millipore, 559389), SP600125 for JNK inhibition (10 μM; Calbiochem, 420119), U0126 for ERK inhibition (10 μM; Millipore, 662005), LY294002 for PI3K/Akt inhibition (1 μM; Millipore, 440202), PD98059 for MEK1/2 inhibition (10 μM; Millipore, 513000), rapamycin for mTOR inhibition (100 nM; Sigma, R0395), CHIR99021 for GSK3β inhibition (10 μM; Tocris, 4423), Y27632 for ROCK inhibition (10 μM; MCE, HY-10583), DAPT for γ-secretase inhibition (25 μM; Sigma, D5942), purmorphamine for SHH activation (2 μM; Sigma, SML0868), and LDN93189 for TGFβ inhibition (100 nM, Cayman, 11802). hBVOs were treated with these compounds at days 15 to 18 of differentiation.

#### Statistical Analysis

Quantitative analysis of the datasets was conducted using GraphPad Prism software. The results are presented as means ± standard error of the mean (SEM).

## Supplementary Information

ESM 1 (DOCX 2950 kb)

## Data Availability

All data that support the finding of this study are available upon request from the corresponding author.
