# Supplementary material for: Blood vessel organoids generated by base editing and harboring single nucleotide variation in Notch3 effectively recapitulate CADASIL-related pathogenesis
Source: Mol Neurobiol. 2024 Apr 9;61(11):9171–83. doi: 10.1007/s12035-024-04141-4 (PMC11496345; doi:10.1007/s12035-024-04141-4)
Supplement: Supplementary file 1 — (DOCX 2950 kb) [file 12035_2024_4141_MOESM1_ESM.docx]

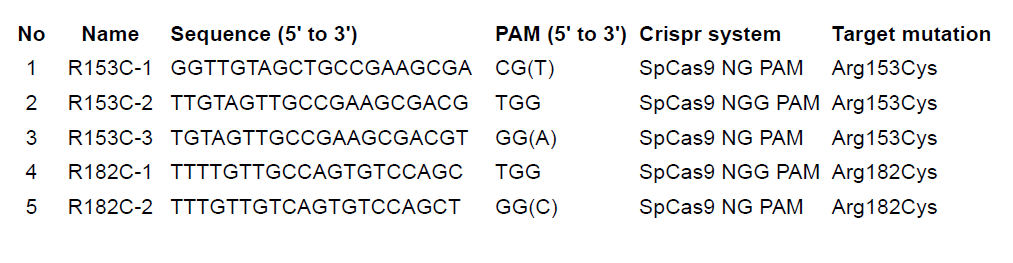


Supplementary Table 1 sgRNA candidates for targeted site recognition in the Notch3 gene. The list presents the selected sgRNA candidates for introducing mutations in the Notch3 gene. Five sgRNAs were chosen, with three sgRNAs designed to induce the R153C mutation, and two sgRNAs to induce the R182C mutation.


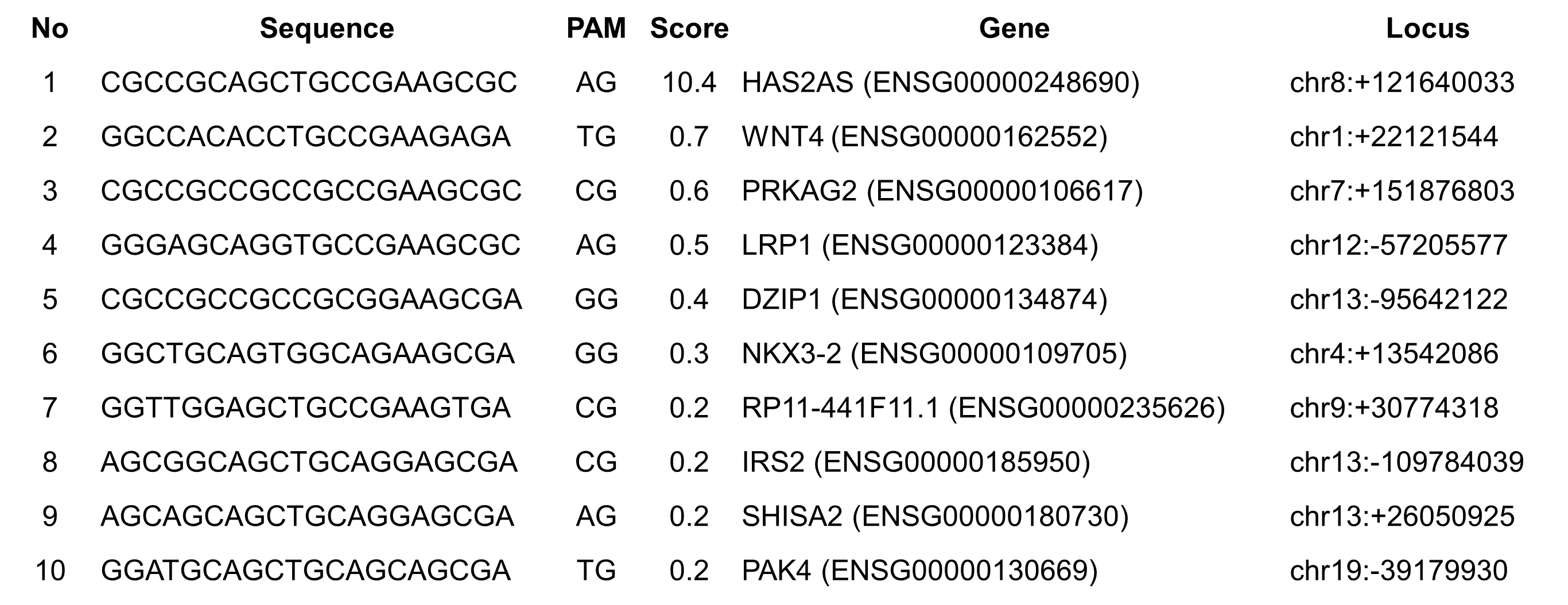


**Supplementary Table 2** Top 10 off-target sites predicted for R153C-1 sgRNA. Top 10 predicted off-target sites associated with the R153C-1 sgRNA. These sites represent genomic locations that may be susceptible to unintended editing by the sgRNA, highlighting the importance of assessing potential off-target effects when using this sgRNA to target the R153C mutation.


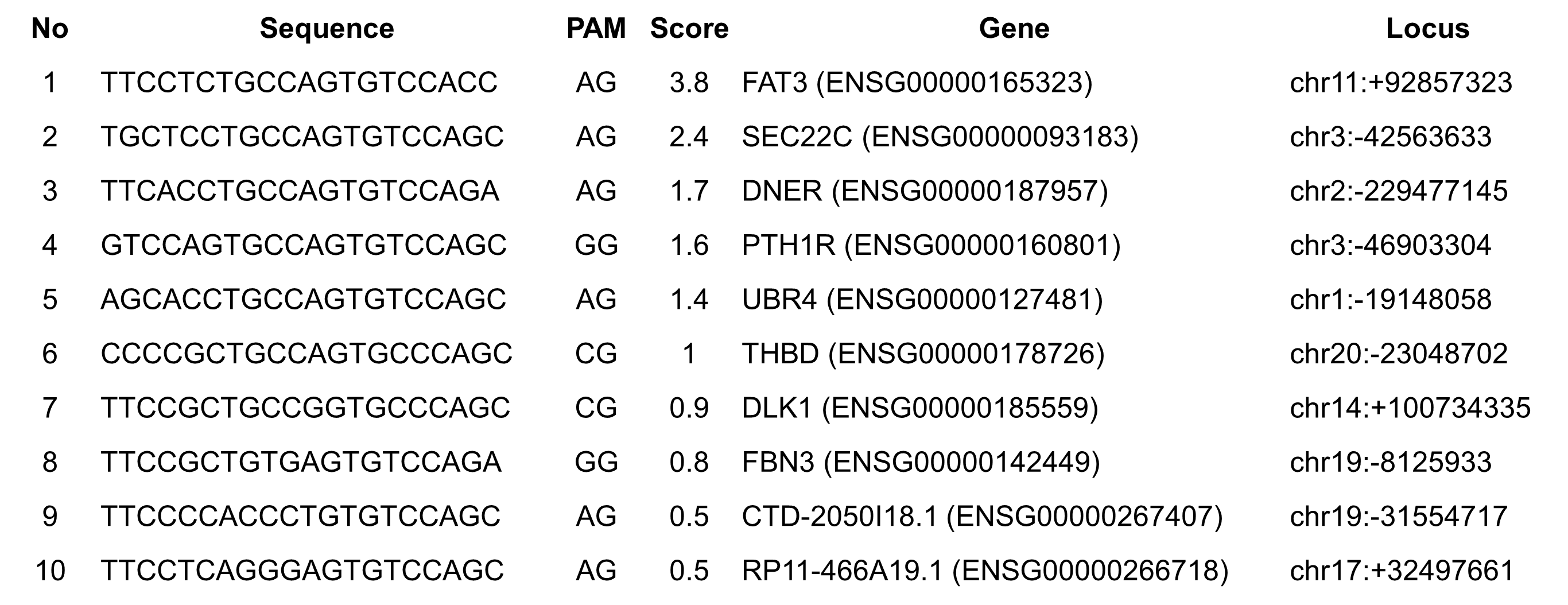


**Supplementary Table 3** Top 10 off-target sites predicted for the R182C-1 sgRNA. Top 10 predicted off-target sites associated with the R182C-1 sgRNA. These sites are genomic locations that may be susceptible to unintended editing by the sgRNA, highlighting the importance of assessing potential off-target effects when using this sgRNA to target the R182C mutation.


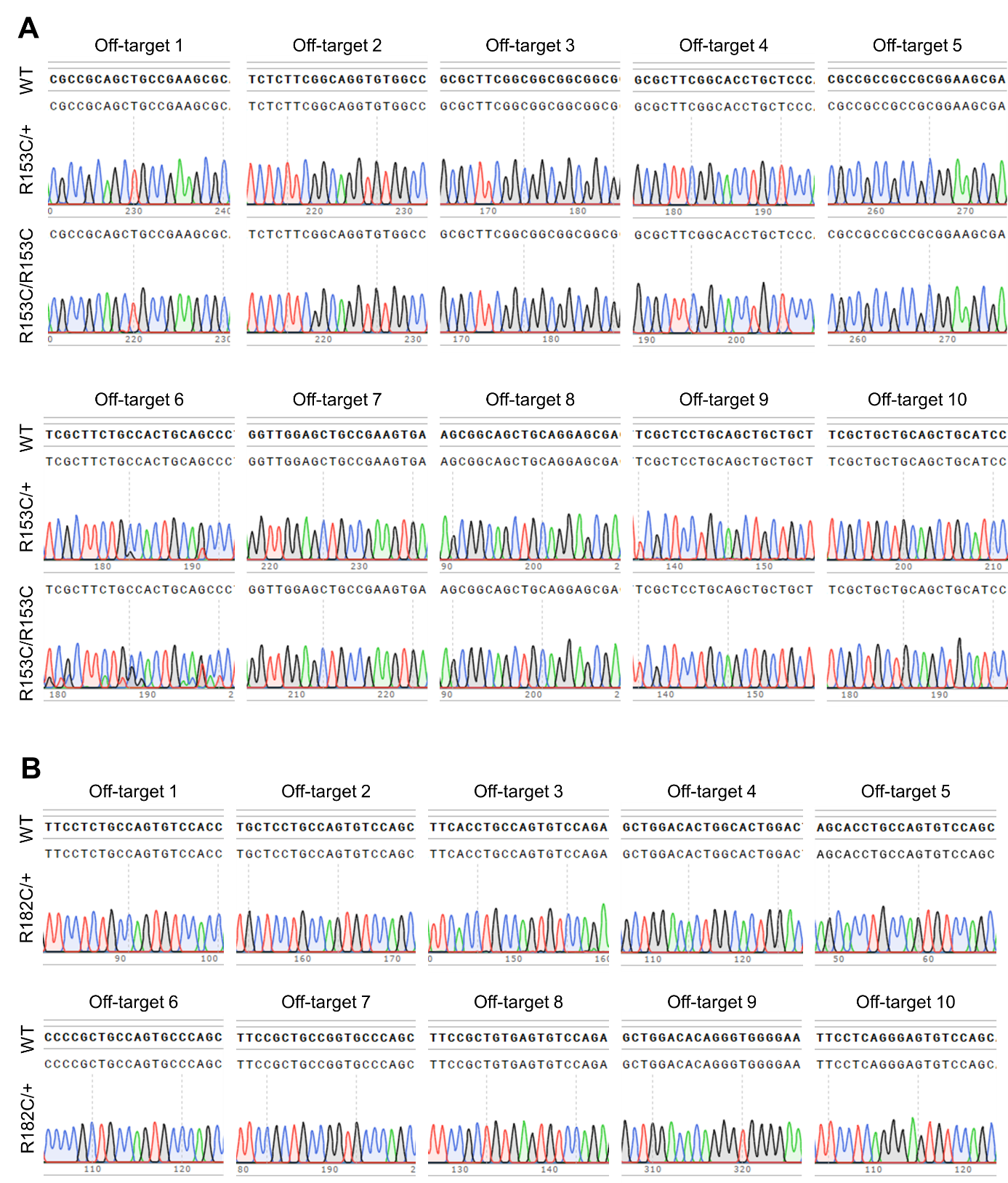


**Supplementary Figure 1** Off-target sequencing in WT and Notch3 mutant hiPSCs. (A) Sanger sequencing of R153C homozygous or heterozygous hiPSCs. (B) Sanger sequencing of R182C heterozygous hiPSCs.


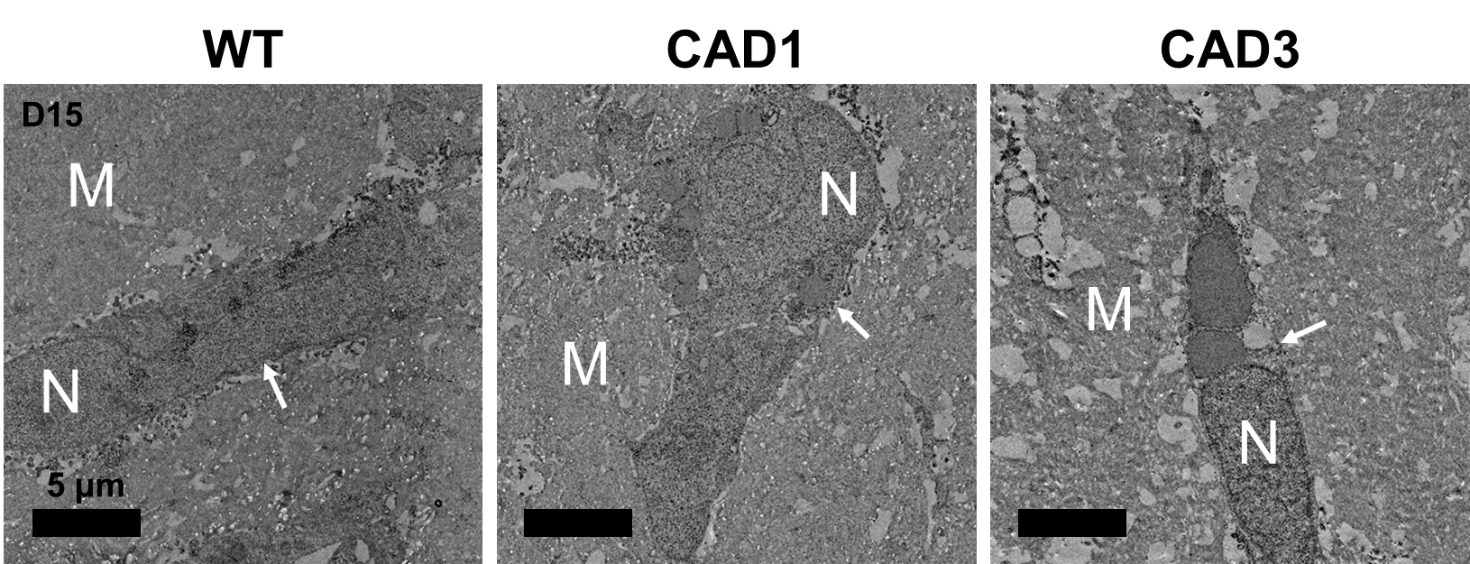


**Supplementary Figure 2** TEM images for Notch3 mutant hBVOs. TEM images were used to detect GOM in Notch3 mutant hBVOs. GOM has not been detected on all specimens including Notch3 mutant hBVOs. Wild type (WT), CAD1 (R153C heterozygous), CAD3 (R182C heterozygous). Scale bar, 5 μm. (Arrow: Endothelial cell, N: Nucleus, M: Matrix)


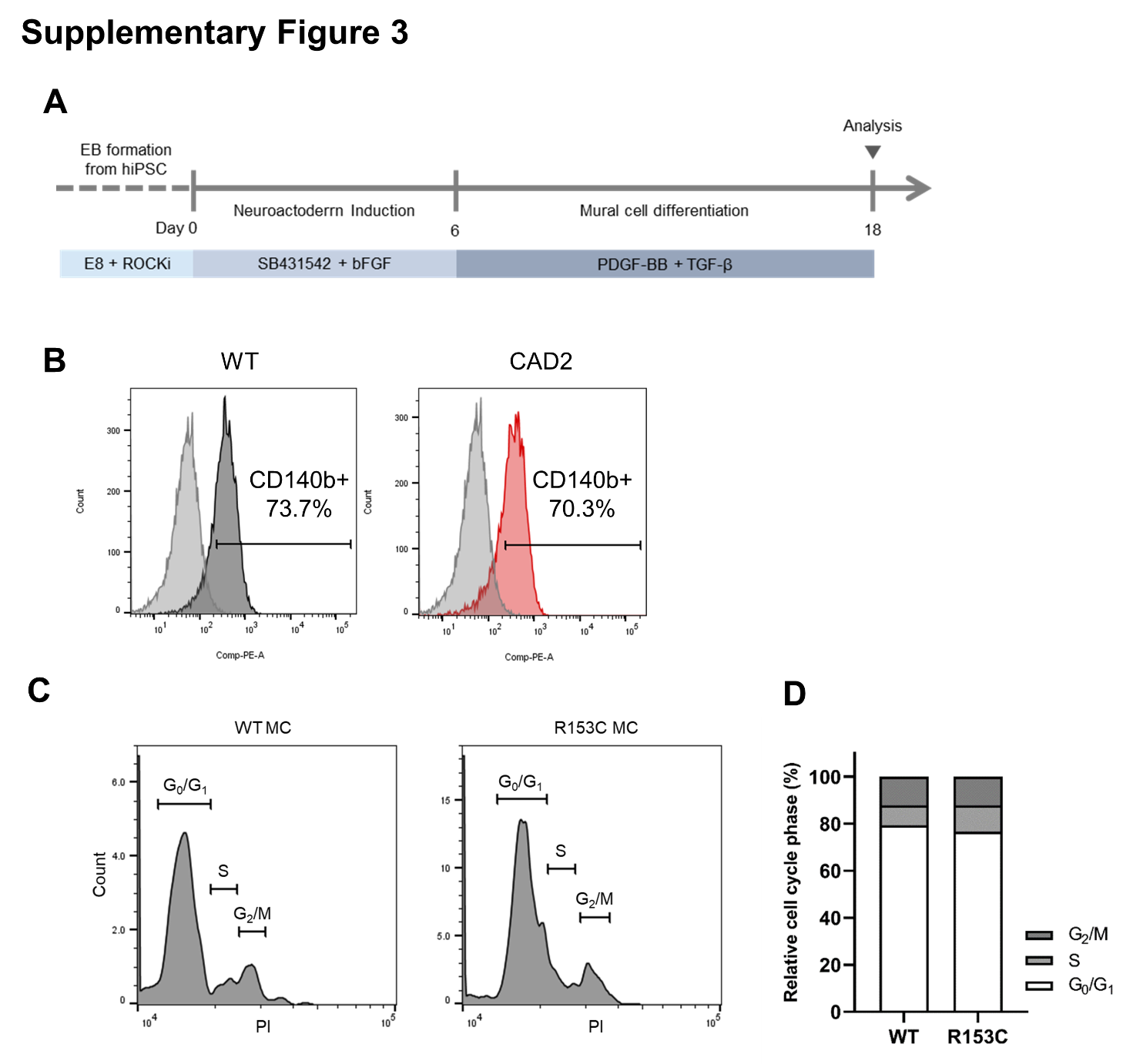


**Supplementary Figure 3** Characteristics of mural cells derived from Notch3 mutant hiPSCs. (A) Schematic of differentiation into mural cells (MC) of wile type (WT) and R153C homozygous Notch3 mutant hiPSCs (R153C/R153C). (B) Proportion of mural cells after differentiation. (C) Flow cytometry histograms of PI staining of WT and R153C homozygous mural cells (right panel). (D) Relative proportions of cells in each cell cycle phase (left panel).
